# Factors influencing long-term health-related quality of life among intensive care unit survivors: a mixed-methods systematic review

**DOI:** 10.3389/fpubh.2026.1919513

**Published:** 2026-09-09

**Authors:** Jie Ou, Yan Wu, Xiaohong Wang, Jiaqi Li

**Affiliations:** 1 Department of Critical Care Medicine, Hainan General Hospital, Affiliated Hainan Hospital of Hainan Medical University Haikou, Haikou, Hainan, China; 2 Department of Nursing, Hainan General Hospital, Affiliated Hainan Hospital of Hainan Medical University Haikou, Haikou, Hainan, China

**Keywords:** health-related quality of life, influence factors, intensive care unit, mixed-methods systematic review, survivors

## Abstract

**Objective:**

To comprehensively synthesize the objective clinical determinants and subjective experiences shaping long-term health-related quality of life (HRQOL) among Intensive Care Unit (ICU) survivors.

**Methods:**

Seven databases were searched through May 1, 2026. Eligible studies examined HRQOL and its determinants in adult ICU survivors. Outcomes assessed at ≥3 months after discharge were used for the primary long-term interpretation, whereas assessments before 3 months were retained as early-recovery evidence. Two reviewers independently screened studies, extracted data, and appraised individual studies using the Mixed Methods Appraisal Tool (MMAT). Quantitative evidence was synthesized by effect direction and major determinant, HRQOL bodies of evidence were assessed using an adapted GRADE approach. Quantitative and qualitative evidence was synthesized separately and subsequently integrated using a convergent segregated approach.

**Results:**

Fifty-three eligible studies were included in the final synthesis. Long-term HRQOL was associated with pre-existing health status, acute illness severity, post-intensive care physical, psychological, and cognitive impairments, functional limitation, rehabilitation, and social support. Pre-ICU chronic disease, frailty, disability, and baseline HRQOL emerged as important contextual determinants and potential confounders of apparent ICU-related associations.

**Conclusions:**

Long-term HRQOL impairment is a multi-layered outcome of biomedical, symptomatic, functional, and environmental interactions. Post-ICU rehabilitation should transition toward multidisciplinary, continuous care that combines physical therapy with cognitive-psychological support and robust transitional care networks to foster successful societal reintegration.

**Systematic review registration:**

https://www.crd.york.ac.uk/PROSPERO/view/CRD420261398874, identifier: CRD420261398874.

## 1 Introduction

Advances in critical care medicine have substantially improved survival among critically ill patients, shifting the focus of care from short-term survival to long-term quality of life. However, increased survival has not necessarily translated into full recovery, resulting in a growing population of survivors with persistent functional impairments (1, 2). Evidence suggests that 50%−70% of Intensive Care Unit (ICU) survivors experience Post-Intensive Care Syndrome (PICS), characterized by physical dysfunction, cognitive impairment, and psychological sequelae (3). Consequently, their health-related quality of life (HRQOL) is often significantly lower than that of the general population (4). As a key indicator of long-term recovery and successful societal reintegration, HRQOL reflects not only individual health outcomes and family caregiving burden but also the broader effectiveness and sustainability of healthcare systems (5). Therefore, identifying the determinants of impaired HRQOL and the mechanisms through which recovery can be promoted has become a priority in critical care rehabilitation and public health research.

However, HRQOL is inherently a complex, multidimensional, and dynamic construct shaped by the interaction of physiological, psychological, social, and individual factors (6). Quantitative studies can estimate associations between clinical or personal characteristics and HRQOL but may not explain how survivors experience symptoms, functional loss, and social disruption. Qualitative studies provide contextual accounts of recovery and support needs, but their transferability is limited by sample size and setting. The two evidence types are therefore complementary: qualitative findings can help explain context and perceived mechanisms, but they do not validate quantitative effect estimates. Although the number of survivor-centered qualitative sources is small, the review retains a mixed-methods designation because it prospectively planned separate quantitative and qualitative syntheses with a formal convergent segregated integration, following established mixed-methods systematic review methodology. The qualitative component, though limited, provides contextual depth that cannot be obtained from quantitative data alone and informs the interpretation of observed associations.

To address these gaps, this study is guided by the Wilson and Cleary model of HRQOL (7) and integrates the PICS (8) with the International Classification of Functioning, Disability and Health (ICF) framework (9). Quantitative evidence on reported associations and qualitative evidence on lived experiences were synthesized separately and then compared to identify convergence, complementarity, divergence, and areas addressed by only one evidence stream. The review sought to distinguish pre-existing health from ICU-associated factors, organize findings by recovery phase, and identify plausible pathways without treating observational associations as causal effects. This review was prospectively registered with PROSPERO (Registration No.: CRD420261398874).

## 2 Methods

### 2.1 Literature search strategy

A comprehensive electronic search was conducted in PubMed, Embase, Web of Science, the Cochrane Library, China National Knowledge Infrastructure (CNKI), Wanfang Database, and China Biomedical Database (CBM) from inception through 1 May 2026. Controlled vocabulary and free-text terms covered “intensive care, survivorship, post-discharge follow-up, HRQOL, and longitudinal outcomes”. Reference lists of included studies were also screened. Eligible designs included quantitative, qualitative, and mixed-methods studies. The PubMed strategy is shown in Box 1; complete database-specific strategies, search dates, limits, and yields are provided in the (Tables 1, 2).

Box 1 Literature search strategy.

1# “Intensive Care Units”[Mesh] OR “Critical Care”[Mesh] OR “Critical Illness”[Mesh] OR “ICU”[tiab]OR “intensive care”[tiab]OR “critical care”[tiab] OR “critical illness”[tiab] OR “intensive care unit^*^”[tiab] OR “post-intensive care”[tiab] OR “post ICU”[tiab] OR “post-ICU”[tiab]2# “Survivors”[Mesh] OR “survivor^*^”[tiab] OR “survivorship”[tiab] OR “after discharge”[tiab] OR “discharge^*^”[tiab] OR “follow-up”[tiab] OR “longitudinal”[tiab]3# “Quality of Life”[Mesh] OR “Health Status”[Mesh] OR “Patient Reported Outcome Measures”[Mesh] OR “quality of life”[tiab] OR “health-related quality of life”[tiab] OR “health related quality of life”[tiab] OR “HRQoL”[tiab] OR “HRQOL”[tiab] OR “QoL”[tiab] OR “QOL”[tiab] OR “health status”[tiab] OR “patient-reported outcome^*^”[tiab] OR “SF-36”[tiab] OR “Short Form-36”[tiab] OR “SF-12”[tiab] OR “Short Form-12”[tiab] OR “EQ-5D”[tiab] OR “EuroQol[tiab] ” OR “WHOQOL”[tiab]

**Table 1:** Search-yield reconciliation.

Source	Platform	Search date	Initial records
PubMed	NLM PubMed	2026-05-01	2963
Embase	Elsevier Embase.com	2026-05-01	1397
Web of Science	Web of Science Core Collection	2026-05-01	582
Cochrane Library	Wiley Cochrane Library	2026-05-01	205
CNKI	China National Knowledge Infrastructure	2026-05-01	160
Wanfang Data	Wanfang Data Knowledge Service Platform	2026-05-01	82
CBM	SinoMed/China Biomedical Literature Database	2026-05-01	109
Other sources	Backward reference-list checking	Up to 2026-05-01	0

**Table 2:** Complete search strategies for all databases.

Database	Search line	Search strategy
Embase	#1	‘intensive care unit'/exp OR ‘critical care'/exp OR ‘critical illness'/exp OR ICU:ti,ab,kw OR ‘intensive care':ti,ab,kw OR ‘critical care':ti,ab,kw OR ‘critical illness':ti,ab,kw OR ‘intensive care unit^*^':ti,ab,kw OR ‘post-intensive care':ti,ab,kw OR ‘post ICU':ti,ab,kw
	#2	‘survivor'/exp OR survivor^*^:ti,ab,kw OR survivorship:ti,ab,kw OR ‘after discharge':ti,ab,kw OR discharge^*^:ti,ab,kw OR ‘follow up':ti,ab,kw OR followup:ti,ab,kw OR longitudinal:ti,ab,kw
	#3	‘quality of life'/exp OR ‘health status'/exp OR ‘patient reported outcome'/exp OR ‘quality of life':ti,ab,kw OR 'health-related quality of life':ti,ab,kw OR ‘health related quality of life':ti,ab,kw OR HRQoL:ti,ab,kw OR HRQOL:ti,ab,kw OR QoL:ti,ab,kw OR QOL:ti,ab,kw OR ‘health status':ti,ab,kw OR ‘patient-reported outcome^*^':ti,ab,kw OR 'SF-36':ti,ab,kw OR ‘SF-12':ti,ab,kw OR ‘EQ-5D':ti,ab,kw OR EuroQol:ti,ab,kw OR WHOQOL:ti,ab,kw
	#4	#1 AND #2 AND #3
	#5	#4 AND [embase]/lim AND [01-01-1947]/sd NOT [02-05-2026]/sd
Web of Science	#1	TS=(“intensive care unit^*^” OR ICU OR “intensive care” OR “critical care” OR “critical illness” OR “post-intensive care” OR “post ICU” OR “post-ICU”)
	#2	TS=(survivor^*^ OR survivorship OR “after discharge” OR discharge^*^ OR “follow-up” OR followup OR longitudinal)
	#3	TS=(“quality of life” OR “health-related quality of life” OR “health related quality of life” OR HRQoL OR HRQOL OR QoL OR QOL OR “health status” OR “patient-reported outcome^*^” OR “SF-36” OR “Short Form-36” OR “SF-12” OR “Short Form-12” OR “EQ-5D” OR EuroQol OR WHOQOL)
	#4	#1 AND #2 AND #3
Cochrane Library	#1	MeSH descriptor: [Intensive Care Units] explode all trees
	#2	MeSH descriptor: [Critical Care] explode all trees
	#3	MeSH descriptor: [Critical Illness] explode all trees
	#4	(ICU OR “intensive care” OR “critical care” OR “critical illness” OR “intensive care unit^*^” OR “post-intensive care” OR “post ICU” OR “post-ICU“):ti,ab,kw
	#5	#1 OR #2 OR #3 OR #4
	#6	MeSH descriptor: [Survivors] explode all trees
	#7	(survivor^*^ OR survivorship OR “after discharge” OR discharge^*^ OR “follow-up” OR followup OR longitudinal):ti,ab,kw
	#8	#6 OR #7
	#9	MeSH descriptor: [Quality of Life] explode all trees
	#10	MeSH descriptor: [Health Status] explode all trees
	#11	(“quality of life” OR “health-related quality of life” OR “health related quality of life” OR HRQoL OR HRQOL OR QoL OR QOL OR “health status” OR “patient-reported outcome^*^” OR “SF-36” OR “SF-12” OR “EQ-5D" OR EuroQol OR WHOQOL):ti,ab,kw
	#12	#9 OR #10 OR #11
	#13	#5 AND #8 AND #12
CNKI and Wanfang	#1	主题= (“重症监护室” OR “重症监护病房” OR “重症医学科” OR “ICU” OR “危重症” OR “危重患者” OR ““重症患者” OR “重症监护”)
	#2	主题= (“幸存者” OR “存活者” OR “生存者” OR ““出院后” OR ““出院” OR “随访” OR “ICU后” OR ““重症后” OR “长期预后“)
	#3	主题= (“健康相关生活质量” OR “健康相关生存质量” OR “健康相关生命质量” OR “生活质量” OR ““生存质量” OR “生命质量” OR “健康状态” OR “SF-36” OR “SF-12” OR “EQ-5D” OR “EuroQol“)
	#4	#1 AND #2 AND #3
CBM/SinoMed	#1	““重症监护病房“[[不加权:扩展] OR “重症监护”[常用字段] OR “重症医学科”[常用字段] OR “ICU”[[常用字段] OR ““危重症”[常用字段] OR “危重患者”[常用字段] OR “重症患者”[常用字段]
	#2	“幸存者”[常用字段] OR “存活者”[常用字段] OR “生存者”[常用字段] OR ““出院后”[常用字段] OR “出院”[常用字段] OR “随访”[常用字段] OR “ICU后”[常用字段] OR “重症后”[常用字段] OR “长期预后”[常用字段]
	#3	“生活质量”[不加权:扩展] OR ““健康相关生活质量”[常用字段] OR “健康相关生存质量”[[常用字段] OR “健康相关生命质量”[常用字段] OR “生存质量”[[常用字段] OR “生命质量”[常用字段] OR “健康状态”[常用字段] OR “SF-36”[常用字段] OR “SF-12”[常用字段] OR “EQ-5D”[常用字段]
	#4	#1 AND #2 AND #3

### 2.2 Inclusion and exclusion criteria

Studies were included if they met the following criteria: (1) participants were adult ICU survivors (≥18 years); (2) the study examined HRQOL and its determinants after ICU care; and (3) the study used a quantitative, qualitative, or mixed-methods design. Studies were excluded if they were duplicate publications, conference abstracts, or unavailable in full text. Thesis and dissertation research indexed in academic databases was eligible for inclusion; other non-peer-reviewed gray literature (e.g., institutional reports, policy documents) was excluded. Because no universally accepted threshold defines long-term outcomes after critical illness, we adopted 3 months after hospital discharge as a pragmatic operational threshold for this review (10, 11). Assessments conducted before 3 months were classified as early-recovery outcomes, those conducted from 3 to 12 months as intermediate follow up outcome, and those conducted after 12 months as extended follow up outcomes. This classification was used to prevent outcomes measured during early convalescence from being interpreted as equivalent to outcomes measured several years after discharge.

### 2.3 Literature screening and data extraction

Literature screening and data extraction were independently performed by two reviewers and subsequently cross-checked. Discrepancies were resolved through discussion or consultation with a third reviewer. Duplicate records were removed using EndNote 21. Titles and abstracts were screened initially, followed by full-text assessment for eligibility. Data extracted from eligible studies included the first author, publication year, country or region, study population, sample size, study design, and reported determinants of HRQOL.

### 2.4 Quality assessment

Two reviewers independently assessed each included study using the design-specific criteria of the Mixed Methods Appraisal Tool (MMAT) (12). Criterion-level judgments were retained to identify study-specific methodological limitations. The number of criteria met is presented descriptively in the study-characteristics table, but it was not treated as a validated overall score or directly converted into a GRADE certainty rating. Disagreements were resolved through discussion or consultation with a third reviewer.

### 2.5 Synthesis of included literature

Because heterogeneity in populations, determinant definitions, HRQOL instruments, effect measures, covariate adjustment, and follow-up periods precluded valid meta-analysis for most determinant–outcome associations, we conducted a structured synthesis without meta-analysis informed by the SWiM reporting guideline (13). Quantitative findings were grouped by determinant domain, specific determinant, HRQOL outcome or domain, and follow-up phase: early (< 3 months), intermediate (3–12 months), and extended (> 12 months). When both adjusted and unadjusted estimates were available, adjusted estimates were prioritized. Each study contributed one direction classification to each determinant, outcome, follow-up stratum. Where a study reported multiple estimates within the same stratum, the most comprehensively adjusted estimate was used; findings were classified as mixed when no single direction could be assigned. Associations were coded as indicating poorer HRQOL, better HRQOL, no clear association, or mixed findings. Vote counting was based on the direction of association rather than statistical significance. Directional consistency was classified using prespecified operational thresholds: high consistency when at least 75% of studies reported the same direction, moderate consistency when 60%~74% reported the same direction, and inconsistent when fewer than 60% reported the same direction. Consistency was not assessed for determinants reported by only one study.

### 2.6 Certainty of quantitative evidence

The certainty of the quantitative evidence was assessed for each major determinant, HRQOL association using the GRADE approach adapted for prognostic-factor evidence. The unit of assessment was the body of evidence contributing to a specific determinant outcome association rather than the individual study. Certainty assessments considered risk of bias, inconsistency, indirectness, imprecision, and publication bias. Where applicable, a large magnitude of association, a dose response gradient, and the likely influence of residual confounding were considered as factors that could increase certainty. Evidence certainty was categorized as high, moderate, low, or very low. Two reviewers conducted the assessments independently, with disagreements resolved through discussion or consultation with a third reviewer.

### 2.7 Qualitative synthesis and reflexivity

Qualitative findings were synthesized separately from quantitative associations. Two reviewers extracted first-order findings (participant quotations or directly reported experiences) and second-order findings (primary authors' interpretations). Findings were coded line by line, compared across reports, grouped into descriptive categories, and developed into higher-order analytical themes. Disagreements were resolved through discussion and re-examination of the source report. A study-by-theme matrix was used to record which study contributed to each theme and to identify themes supported by only one source. Divergent findings were retained. Initial coding was inductive; the Wilson and Cleary (7), PICS (8), and ICF frameworks (14) were applied during interpretation rather than used to predetermine all codes. Given the small evidence base, thematic saturation was neither assumed nor claimed.

### 2.8 Mixed-methods integration

Quantitative and qualitative syntheses were compared using a convergent segregated approach. Integration focused on whether the evidence streams converged, complemented one another, diverged, or addressed different aspects of recovery. Qualitative accounts were used to contextualize how associations were experienced and were not treated as validation of quantitative estimates or evidence of causality.

## 3 Results

### 3.1 Literature search results and study inclusion

**Figure 1:**
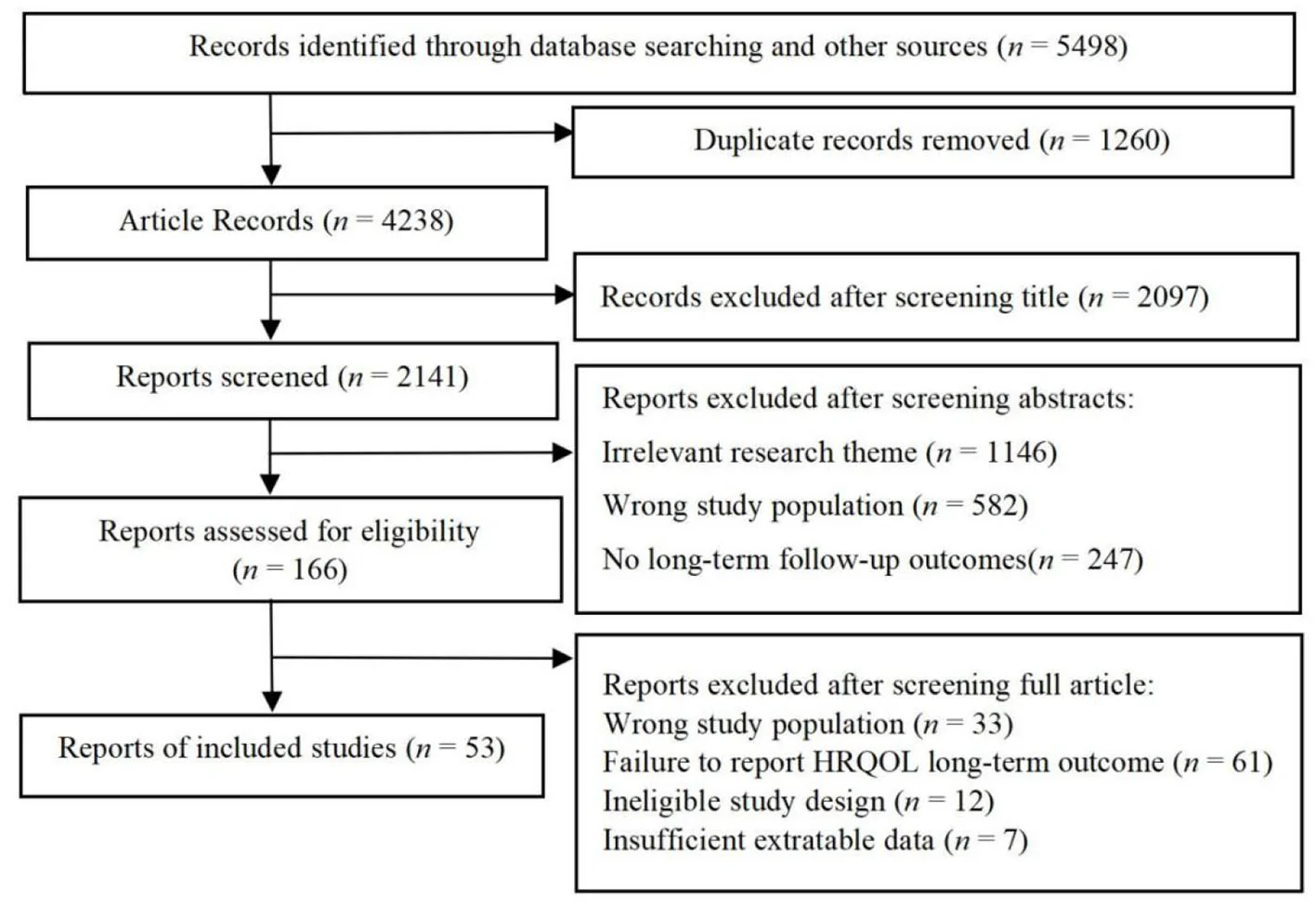
PRISMA 2020 flow diagram of the literature search and study selection process.

A total of 53 studies were included in the final analysis; the literature search and study selection process are illustrated in Figure 1. The studies were conducted across multiple countries including China, the United States, France, Spain, Sweden, and South Korea, with several multinational collaborative investigations. Cohort studies constituted the main study design, alongside cross-sectional studies, case-control studies, qualitative studies and mixed-methods studies. The sample sizes of included studies varied widely, ranging from 14 to 6093 participants. Outcome measurement tools mainly comprised the SF series questionnaires such as SF-36, SF-12 and RAND-36, as well as the EQ-5D series scales. Qualitative studies collected data via semi-structured interviews. There was marked heterogeneity in follow-up time points, covering time points at ICU discharge, hospital discharge, 1, 3, 6, 12, 24, 36 months after discharge, up to 5 years, and a mean of 8 years. Several studies adopted longitudinal follow-up with multiple time points, while a small number of studies failed to clearly report assessment timing. Methodological quality of literature was evaluated using the Mixed Methods Appraisal Tool (MMAT), with scores ranging from 3/5 to 5/5. Most studies achieved an MMAT score of 5, indicating generally good methodological quality of the included literature.

### 3.2 Basic characteristics of the included studies

The characteristics and criterion-level MMAT appraisal of the 53 included studies are summarized in Table 3. Quantitative effect directions are summarized in Table 4, and GRADE certainty judgments for major determinant HRQOL associations are presented in Table 5.

**Table 3:** General characteristics of the included studies (*n* = 53).

References	Country	Research method	Sample size	Influencing factors	Evaluation Tool	Evaluation Nodes	MMAT Score
González-Pérez et al. (31)	USA	Cohort study	205	Age; Severity of illness; Comorbidities; Anxiety; Depression; Nutritional status; Pain; PICS; Physical function	EQ-5D	/	4/5
Fernández-Gonzalo et al. (59)	Spain	Cohort study	142	Age; Gender; Severity of illness; BMI; Muscle weakness; Anxiety; Depression; Pain; PICS; Physical function; COVID-19; Trauma; Respiratory failure; Stress; Cognitive function; Sleep; Rehabilitation time; PTSD	SF-12	1, 3, 6, 9, 12 months	5/5
Lasocki et al. (20)	France	Cohort study	1161	Age; Gender; Comorbidities; Duration of ICU stay; Muscle weakness; Pain; PICS; Rehabilitation time; Psychological factors	SF-36	One year	5/5
Puthucheary et al. (21)	Germany	Cohort study	291	Age; Gender; Severity of illness; Comorbidities; Duration of ICU stay; Mechanical ventilation; BMI; Tracheostomy; Physical function; Rehabilitation time; Tracheostomy tube removal; ADL; Psychological factors; Family support	SF-36	24 months	3/5
Thiolliere et al. (32)	France	Cohort study	19	Age; Gender; Severity of illness; Duration of ICU stay; Duration of hospital stay; Mechanical ventilation; Duration of mechanical ventilation; BMI; Sedation; Muscle weakness; Anxiety; Pain; Physical function; COVID-19; Trauma; Stress; Cognitive function; ADL; Social support; Psychological factors; Position status; Muscle mass; Appetite	EQ-5D	180 days	5/5
Ehooman et al. (33)	France; Belgium	Cohort study	1011	Age; Gender; Severity of illness; Comorbidities; Mechanical ventilation; Tracheostomy; Sedation; Muscle weakness; Anxiety; Depression; CRRT; Pain; Physical function; Stress; PTSD; Psychological factors; Position status; Organ failure; Underlying acute disease duration	SF-36	3 months; 1 year	5/5
Demoule et al. (34)	France	Cohort study	42	Age; Gender; Severity of illness; Comorbidities; Duration of hospital stay; Mechanical ventilation; BMI; Muscle weakness; Anxiety; Depression; CRRT; Stress; Rehabilitation time; Tracheostomy tube removal; ADL; Social support; Muscle mass; Exercise capacity	EQ-5D	2 and 12 months	5/5
Lobo-Valbuena et al. (35)	Spain	Cohort study	1462	Age; Gender; Severity of illness; Duration of ICU stay; Mechanical ventilation; Tracheostomy; Sedation; Muscle weakness; Pain; Physical function; Trauma; Cognitive function; ADL; Family support; Delirium; Memory; Adaptation	SF-36	Upon ICU admission, ICU discharge, hospital discharge, and 2-year follow-up	4/5
Halvorsen et al. (24)	Sweden	Cohort study	252	Age; Gender; Severity of illness; Comorbidities; Duration of ICU stay; Duration of hospital stay; Mechanical ventilation; BMI; Muscle weakness; Anxiety; Pain; Physical function; COVID-19; Respiratory failure; Cognitive function; Rehabilitation time; Psychological factors; Underlying acute disease duration (36)	RAND-36	At discharge 3~6 months	4/5
Amacher et al. (27)	Switzerland	Cohort study	46	Age; Gender; Severity of illness; Comorbidities; Duration of ICU stay; Anxiety; Depression; Pain; PICS; Physical function; Delirium; Psychological compliance; PTSD; Trauma; Stress; Rehabilitation time; Social support; Personality type	EQ-5D-3L	24 months	5/5
Kim et al. (37)	South Korea	Cohort study	238	Age; Gender; Severity of illness; Comorbidities; Duration of ICU stay; Duration of hospital stay; Mechanical ventilation; BMI; Muscle weakness; Anxiety; Depression; Pain; Cognitive function; Psychological factors; Organ failure; Delirium	SF-36; EQ-5D-5L	30 and 90 days after discharge	5/5
Ersson et al. (38)	Sweden	Cohort study	41	Age; Gender; Severity of illness; Comorbidities; Duration of hospital stay; Mechanical ventilation; BMI; Tracheostomy; Sedation; Muscle weakness; Anxiety; Depression; Pain; Trauma; Stress; Cognitive function; Sleep; Rehabilitation time; Tracheostomy tube removal; ADL; Organ failure; Exercise capacity; Delirium; Personality type	RAND-36	2,6,12 months	5/5
Abraham et al. (39)	USA	Cross-sectional study	173	Age; Gender; Severity of illness; Comorbidities; Duration of ICU stay; Mechanical ventilation; BMI; Depression; Pain; Physical function; Trauma; Stress; PTSD; Delirium; Education Level; Employment status	SF-36	1 year	5/5
Scheunemann et al. (15)	England	Qualitative study	39	Age; Gender; Severity of illness; Comorbidities; Duration of hospital stay; Pain; Physical function; Trauma; Stress; Rehabilitation time; Tracheostomy tube removal; ADL; Social support; Family support; Psychological factors; Underlying acute disease duration; Adaptation; Continuity of medical care	Semi-structured Interview	ICU transition to wards, early period after discharge to home, and late period after discharge to home	4/5
He et al. (22)	China; USA	Cohort study	483	Age; Gender; Severity of illness; Comorbidities; Duration of ICU stay; Mechanical ventilation; Duration of mechanical ventilation; Anxiety; Depression; CRRT; Pain; Respiratory failure; Cognitive function; ADL; Position status; Organ failure	EQ-5D	1 year	5/5
Busico et al. (60)	Argentina	Cohort study	110	Age; Severity of illness; Comorbidities; Duration of ICU stay; Mechanical ventilation; Duration of mechanical ventilation; Sedation; Physical function; Stress; Cognitive function; ADL; Social support; Family support; Psychological factors; Delirium; Follow-up interval	EQ-5D	At 3 and 12 months after discharge	5/5
Granja et al. (40)	Portugal	Cohort study	464	Age; Gender; Severity of illness; Duration of ICU stay; Duration of hospital stay; Mechanical ventilation; Sedation; Anxiety; Depression; Pain; Physical function; Trauma; Stress; Cognitive function; Fatigue; PTSD; Social support; Family support	EQ-5D; SF-36	Six months	5/5
Ylipalosaari et al. (61)	Finland	Cohort study	272	Age; Gender; Severity of illness; Duration of ICU stay; Duration of hospital stay; Tracheostomy; Anxiety; Depression; Pain; Physical function; Stress; ADL; Family support; Organ failure; Underlying acute disease duration; Employment status	EQ-5D	24 months	5/5
Orwelius et al. (18)	Sweden	Cross-sectional study	5306	Age; Gender; Severity of illness; Comorbidities; Duration of hospital stay; Tracheostomy; Pain; Physical function; Trauma; ADL; Social support; Underlying acute disease duration; Education Level; Employment status; Continuity of medical care	EQ-5D; SF-36	36 months	5/5
Ferrand et al. (62)	France	Cohort study	170	Age; Gender; Severity of illness; Duration of ICU stay; Duration of hospital stay; Mechanical ventilation; Duration of mechanical ventilation; Tracheostomy; Muscle weakness; Anxiety; Depression; CRRT; Nutritional status; Pain; Physical function; Trauma; Stress; Cognitive function; Sleep; Psychological factors; Organ failure	SF-36	6 months	5/5
Gamberini et al. (58)	Italy	Cohort study	205	Age; Gender; Severity of illness; Comorbidities; Duration of ICU stay; Duration of hospital stay; Mechanical ventilation; Duration of mechanical ventilation; Muscle weakness; Anxiety; Depression; Stress; Cognitive function; Sleep; Tracheostomy tube removal; ADL; Delirium; Continuity of medical care	SF-36	90 days	5/5
Griffith et al. (19)	England	Cohort study	240	Age; Gender; Severity of illness; Comorbidities; Mechanical ventilation; Duration of mechanical ventilation; Pain; Physical function; Rehabilitation time; Fatigue; Social support; Psychological factors; Organ failure; Exercise capacity	SF-36; SF-12; HUI	12 months	5/5
Heyland et al. (63)	Canada	Cohort study	610	Age; Gender; Severity of illness; Comorbidities; Duration of hospital stay; Mechanical ventilation; Duration of mechanical ventilation; BMI; Muscle weakness; Physical function; Family support; Psychological factors; Underlying acute disease duration	SF-36	12 months	5/5
Li et al. (41)	China	Cohort study	112	Family support; Financial burden; Personality type; Respiratory related complications; Extensive trauma	SF-36	6 months	4/5
Peng et al. (42)	China	Cross-sectional study	218	Age; Severity of illness; Sputum suction frequency; Marriage; Cognitive function; Delirium	EQ-5D-5L	1 month	5/5
Yao et al. (30)	China	Cross-sectional study	128	Age; Duration of ICU stay; Duration of hospital stay	SF-36	6 months	5/5
Gu et al. (56)	China	Case-control study	79	Age; Severity of illness; Duration of ICU stay	SF-36	1 month	5/5
He et al. (43)	China	Cross-sectional study	253	Age; Gender; Education Level	SF-36	1 month	4/5
Neelima and Chivukula (44)	India	Cross-sectional study	107	Age; Gender; Comorbidities; Duration of ICU stay; Education Level	EQ-5D-5L	1 month	4/5
Kang et al. (64)	South Korea	Cross-sectional study	534	Age; Gender; Severity of illness; Duration of ICU stay; Employment status	EQ-5D-5L	12 months after discharge	5/5
Khoudri et al. (45)	Morocco	Cohort study	145	Age; Gender; Severity of illness; Comorbidities; Duration of ICU stay; Mechanical ventilation; Duration of mechanical ventilation	SF-36	3 months	4/5
Pastene et al. (25)	France; Belgium	Cohort study	2087	Age; Gender; Severity of illness; Comorbidities; Duration of ICU stay; Mechanical ventilation; Duration of mechanical ventilation; BMI; Stress; Cognitive function; PTSD; Tracheostomy tube removal; Organ failure; Social support	SF-36	1 year	5/5
Gamberini et al. (23)	Italy	Cohort study	178	Age; Gender; Severity of illness; Comorbidities; Anxiety; Depression; Pain; Respiratory failure; Stress; Sleep; Organ failure	15D instrument	1 year	5/5
McNelly et al. (46)	England	Cohort study	91	Age; Gender; Comorbidities; Pain; Physical function; Stress; Cognitive function; Rehabilitation time; Social support; Underlying acute disease duration	SF-36	18 months	4/5
Myhren et al. (47)	Norway	Cohort study	194	Age; Gender; Severity of illness; Comorbidities; Duration of ICU stay; Anxiety; Depression; Cognitive function; Rehabilitation time; Social support; Appetite; Memory; Education Level; Employment status	SF-36	1 year	5/5
Niittyvuopio et al. (48)	Finland	Cohort study	351	Pain; Delirium; Memory	SF-36	3 months	4/5
Orwelius et al. (36)	Sweden	Cohort study	6093	Age; Gender; Severity of illness; Comorbidities; Self-care ability	SF-36	At hospital	5/5
Orwelius et al. (36)	Sweden	Cohort study	980	Age; Gender; Severity of illness; Comorbidities; Anxiety; Depression; Pain; Physical function; Trauma; Sleep; Underlying acute disease duration; Exercise capacity; Education Level; Continuity of medical care; Recurrence	SF-36	6, 12, 24, and 36 months	5/5
Pfoh et al. (49)	USA	Cohort study	193	Age; Gender; Severity of illness; Comorbidities; Duration of ICU stay; Duration of hospital stay; Mechanical ventilation; Duration of mechanical ventilation; Sedation; Physical function; Rehabilitation time; Organ failure; Exercise capacity; Delirium; Follow-up interval	SF-36	3,6 months	5/5
van Vliet et al. (65)	Netherlands	Cohort study	34	Age; Gender; Severity of illness; Underlying acute disease duration; Duration of mechanical ventilation; Duration of hospital stay; Muscle weakness; Fatigue; Anxiety; Depression; Stress; Cognitive function; Memory; Psychological factors; Social support; Family support	SF-36	In hospital 18 months	4/5
Nannan Panday et al. (66)	Netherlands	Cohort study	880	Multi-center research	SF-36	28 days	5/5
Kim et al. (50)	South Korea	Cohort study	121	Age; Gender; Severity of illness; Duration of hospital stay; Sedation; Muscle weakness; Anxiety; CRRT; Pain; Stress; Cognitive function; Rehabilitation time; PTSD; ADL; Position status; Delirium; Education Level; Marriage (36)	EQ-5D-5L	5 years	5/5
Kurematsu and Ikematsu (67)	Japan	Cohort study	81	Age; Gender; Severity of illness; Duration of ICU stay; Tracheostomy; Physical function; Trauma; Stress; Cognitive function; Rehabilitation time; ADL; Family support; Psychological factors	HRQoL-20	At ICU discharge; hospital discharge; one month after discharge	5/5
Ringdal et al. (51)	Sweden	Cohort study	239	Age; Gender; Severity of illness; Duration of ICU stay; Duration of hospital stay; Mechanical ventilation; Duration of mechanical ventilation; BMI; Sedation; Anxiety; Depression; Pain; Physical function; Stress; Social support; Family support; Psychological factors; Organ failure; Underlying acute disease duration; Delirium; Memory	SF-36	At discharge 6,18 month	5/5
Van der Merwe et al. (52)	South Africa	Cohort study	107	Age; Gender; Severity of illness; Duration of ICU stay; Duration of hospital stay; Mechanical ventilation; Duration of mechanical ventilation; Tracheostomy; Sedation; Anxiety; Depression; Pain; Physical function; COVID-19; Trauma; Cognitive function; Rehabilitation time; PTSD; Social support; Organ failure; Delirium; Employment status	SF-36	At discharge 6 weeks and 6 months	5/5
Schelling et al. (53)	Germany	Cohort study	148	Age; Gender; Severity of illness; Anxiety; Depression; Pain; Physical function; Respiratory failure; Stress; Cognitive function; PTSD; Memory	SF-36	At discharge 6 months	5/5
Timmers et al. (54)	Netherlands	Cohort study	834	Age; Gender; Severity of illness; Duration of ICU stay; Duration of hospital stay; Mechanical ventilation; Duration of mechanical ventilation; Anxiety; Depression; Pain; Physical function; Trauma; Cognitive function; Marriage	EQ-6D	Mean follow up 8 years	5/5
Ulvik et al. (68)	Norway	Cohort study	228	Age; Gender; Severity of illness; Duration of ICU stay; Tracheostomy; Pain; Organ failure; Underlying acute disease duration	EQ-5D	2~7 years	5/5
Li et al. (55)	China	Cohort study	156	Duration of ICU stay; Pain	SF-36	At discharge 3, 6, 12 months	5/5
Lin et al. (26)	China	Cohort study	285	Gender; Severity of illness; Comorbidities; Duration of mechanical ventilation; Follow-up interval	SF-36	At discharge 3, 6 months	5/5
Zhang (16)	China	Mixed Methods study	16/136	Severity of illness; Comorbidities; Anxiety; Depression; Pain; ADL; Social support; Family support; Education Level; Employment status; Condition at admission; Fear of movement; Uncertainty of illness	SF-36	/	4/5
Li et al. (28)	China	Cohort study	67	Age; Depression; Status of caregiver; Compliance; Professional support	SF-36	At discharge	5/5
Hao et al. (29)	China	Cross-sectional study	150	Age; Severity of illness; Duration of ICU stay; Duration of hospital stay; Duration of mechanical ventilation; BMI; Tracheostomy; Pain; Physical function; Cognitive function; Rehabilitation time; ADL; Position status; Delirium; Memory; Education Level (65)	SF-36	At discharge 3 months	5/5

ICU, intensive care unit; HRQOL, health-related quality of life; MMAT, Mixed Methods Appraisal Tool. MMAT results are descriptive criterion-level summaries and are not treated as validated overall quality scores.

**Table 4:** Direction and consistency of associations between determinants and HRQOL among ICU survivors.

Domain	Factor	Follow-up	Poorer HRQOL direction	Better HRQOL direction	Null/mixed	Consistency	Adjustment status
Baseline health	Pre-ICU HRQOL/functional	3~12; > 12 months	Worse pre-ICU HRQOL or functional status is associated with poorer follow-up HRQOL (18, 26, 49, 54)	Better baseline function correlates with superior HRQOL at follow-up (26, 49)	No consistent opposing evidence	High consistency	Predominantly adjusted; baseline sometimes retrospective
	Comorbidity burden	3~12; > 12 months	Higher comorbidity burden is associated with poorer HRQOL (18, 19, 21, 45, 49)	-	Associations attenuated after adjustment in several studies	Moderate consistency	4/5 adjusted
Demographic	Older age	< 3; 3–12; >12 months	Older age was associated with poorer physical HRQOL or less favorable recovery in several cohorts (22, 37, 42, 43, 56)	-	Associations with psychological HRQOL are inconsistent; independent associations absent in some studies	Moderate consistency	Predominantly adjusted
	Female	< 3; 3–12; >12 months	Female sex correlates with poorer scores in selected physical or overall HRQOL domains (37–39, 52)	-	Multiple studies failed to identify independent associations, or reported effects limited to specific domains	Inconsistent	Mixed adjustment
Socioeconomic	Low education attainment	< 3; >12 months	Lower educational attainment is associated with poorer HRQOL and persistent functional impairment (16, 21, 43)	Higher education predicts more favorable recovery (44, 47, 49)	Independent associations not explicitly reported in several investigations	Moderate consistency	Adjusted evidence prioritized
	Unemployment/not returning to work	3-12 months	Unemployment or failure to return to work is associated with poorer HRQOL (32, 47)	-	Employment status may represent an HRQOL outcome rather than an independent predictor	High consistency	Mixed; possible outcome overlap
Acute illness	Greater illness severity	< 3; 3–12 months	Higher acute illness severity is associated with poorer HRQOL (29, 42, 45, 56)	-	Weak or non-significant associations observed after adjustment (19, 23)	Inconsistent	Adjusted evidence prioritized
	Organ failure/active disease status	3-12 months	Organ failure or ongoing disease status correlates with poorer HRQOL (29, 33)	-	Heterogeneity exists across varying definitions of organ failure	Moderate consistency	Adjusted
	Pulmonary infection/pulmonary sepsis	>12 months	Pulmonary infection or pulmonary sepsis is associated with poorer long-term HRQOL (22)	-	-	Not assessed—single study	Adjusted; single study
ICU exposure	Longer ICU stay	< 3; 3–12 months	Prolonged ICU length of stay is associated with poorer HRQOL (29, 30, 44, 56)	-	-	High consistency	3/4 adjusted
	Longer mechanical ventilation	3–12 months	Extended mechanical ventilation correlates with poorer HRQOL (24, 26, 29)	-	Griffith et al. (19) detected no independent association	Inconsistent	Adjusted evidence prioritized
	Delirium	< 3; >12 months	ICU delirium is associated with poorer HRQOL (35, 37, 42)	-	Abraham et al. (39) found no independent link between in-hospital delirium and 1-year HRQOL	Inconsistent	Adjusted
	Tracheostomy	3–12 months	Tracheostomy is associated with poorer HRQOL and restricted recovery (25, 32)	-	Other studies reported no consistent independent association	Inconsistent	Mixed adjustment
Biological	Iron deficiency at ICU	>12 months	Severe iron deficiency correlates with impaired physical recovery and poorer HRQOL at 1 year (20)	-	-	Not assessed—single study	Adjusted; single study
Treatment	Red-blood-cell Transfusion exposure	3-12 months	Greater red blood cell transfusion exposure is associated with poorer HRQOL (31)	-	-	Not assessed—single study	Adjusted incl. propensity score
Physical function	Muscle weakness/ICU acquired weakness	3–12 months; >12 months	ICU-acquired weakness and reduced muscle strength are associated with poorer HRQOL (21, 26, 49)	-	-	High consistency	Predominantly adjusted
	ADL dependence/reduced self-care	< 3; 3–12 months	Dependence in ADL or reduced self-care capacity correlates with poorer HRQOL (29, 36)	-	-	High consistency	Adjusted; temporality limited
	Reduced exercise capacity/physical activity	3–12 months; >12 months	Diminished exercise capacity and low physical activity are associated with poorer HRQOL (46, 48, 49)	-	-	High consistency	Mixed adjustment
Symptoms	Pain	3–12 months; >12 months	Higher pain intensity correlates with poorer HRQOL (48, 55)	-	Independent associations not identified in several studies	Moderate consistency	One adjusted; one correlational
Psychological	Anxiety and/or depression	< 3; 3–12; >12 months	Symptoms of anxiety and depression are associated with poorer HRQOL (37, 47, 50, 52, 53)	-	Associations sometimes restricted to psychological HRQOL domains	High consistency	Predominantly adjusted
	PTSD symptoms/traumatic stress	3–12; >12 months	PTSD symptoms and traumatic stress correlate with poorer HRQOL (39, 40, 47, 51, 53)	-	Heterogeneity stemming from varied PTSD assessment tools and cut-off thresholds across studies	Moderate consistency	Mixed adjustment
Cognitive	Cognitive impairment	< 3; >12 months	Cognitive dysfunction is associated with poorer HRQOL (42, 50)	-	Independent associations absent in some studies	Moderate consistency / limited evidence	Adjusted evidence prioritized
Sleep	Sleep/circadian disturbance	>12 months	Poor sleep quality and circadian disruption are associated with poorer HRQOL (48, 55)	-	Associations partially mediated by anxiety, depression and illness severity	Not assessed	Insufficient independently interpretable evidence
Psychosocial	Poor coping/hopelessness	>12 months	Maladaptive coping and hopelessness correlate with poorer HRQOL (57)	-	-	Not assessed—single study	Adjusted; single study
	Low family/social support	3–12 months	Insufficient family or social support is associated with poorer HRQOL (16, 28)	-	Qualitative studies excluded as quantitative evidence for directional effects	Not assessed - limited quantitative evidence (2 studies)	Limited adjusted quantitative evidence
Behavior	Low self-management/health-behavior capacity	3–12 months	Inadequate self-management and limited health behavior capacity correlate with poorer HRQOL (28)	-	Sparse literature; heterogeneity in conceptual frameworks and measurement tools	Not assessed—single study	Adjusted; single study
Diagnosis	COVID-19 vs. other ICU diagnoses	3–12 months	-	-	ICU admission for COVID-19 was not independently associated with worse HRQOL compared with non-COVID ICU survivors (34); Gamberini et al. (58) are affected by population and comparator group differences	Inconsistent	Adjusted comparison

HRQOL, health-related quality of life; ICU, intensive care unit; ADL, activities of daily living; PTSD, post-traumatic stress disorder. Follow-up was categorized as early (< 3 months), intermediate (3–12 months), and extended (>12 months). Direction coding was based preferentially on multivariable-adjusted estimates rather than statistical significance. Each study contributed only once to a given factor–outcome–follow-up stratum. Studies that measured a factor without reporting an interpretable association were not counted as supporting evidence. Qualitative studies were not included in quantitative effect-direction counts.

**Table 5:** GRADE evidence profile for major determinant–HRQOL associations.

Association	Studies	Risk of bias	Inconsistency	Indirectness	Imprecision	Publication bias	Certainty	Main rationale	Contributing studies
Pre-ICU HRQOL/function → later HRQOL	4	Serious	Not serious	Not serious	Serious	Undetected	Low	Observational evidence; baseline measurement often retrospective or incomplete.	(18, 26, 49, 54)
Comorbidity burden → poorer HRQOL	5	Serious	Not serious	Not serious	Not serious	Undetected	Moderate	Replicated adjusted association and large samples; residual confounding remains.	(18, 19, 21, 45, 49, 54)
Older age → poorer HRQOL	5	Serious	Serious	Not serious	Not serious	Undetected	Low	Direction varied by physical vs. mental domains and follow-up.	(22, 37, 42, 43, 56)
Female sex → poorer HRQOL	4	Serious	Serious	Not serious	Serious	Undetected	Very low	Domain-specific and incompletely adjusted estimates.	(37–39, 52)
Illness severity → poorer HRQOL	6	Serious	Serious	Not serious	Serious	Undetected	Very low	Conflicting adjusted findings; severity measures varied.	(19, 23, 29, 42, 45, 56)
Longer ICU stay → poorer HRQOL	4	Serious	Serious	Serious	Serious	Undetected	Very low	LOS is a proxy for illness/treatment burden and is highly confounded.	(24, 29, 44, 56)
Longer ventilation → poorer HRQOL	4	Serious	Serious	Not serious	Serious	Undetected	Very low	Conflicting direction and heterogeneous exposure definitions.	(19, 26, 29, 30)
Delirium → poorer HRQOL	4	Serious	Serious	Not serious	Serious	Undetected	Very low	Two adverse associations but one adjusted null study; different populations/times.	(35, 37, 39, 42)
Muscle weakness/function loss → poorer HRQOL	3	Serious	Not serious	Not serious	Serious	Undetected	Low	Consistent direction; overlap between exposure and physical HRQOL creates measurement-related concern.	(21, 26, 49)
ADL dependence → poorer HRQOL	2	Serious	Not serious	Serious	Serious	Undetected	Very low	Concurrent/near-concurrent measurement limits temporality.	(29, 36)
Pain → poorer HRQOL	2	Serious	Not serious	Not serious	Serious	Undetected	Low	Consistent longitudinal direction, but one analysis was correlational.	(48, 55)
Anxiety/depression → poorer HRQOL	5	Serious	Not serious	Not serious	Serious	Undetected	Low	Consistent across cohorts; concurrent symptom and HRQOL measurement may inflate association.	(37, 47, 50, 52, 53)
PTSD/stress → poorer HRQOL	5	Serious	Not serious	Not serious	Serious	Undetected	Low	Consistent direction with heterogeneous instruments and adjustment.	(39, 40, 47, 51, 53)
Cognitive impairment → poorer HRQOL	2	Serious	Not serious	Serious	Serious	Undetected	Very low	Few studies; self-report and timing differed.	(42, 50)
Family/social support → better HRQOL	2	Serious	Not applicable	Serious	Serious	Unsuspected	Very low	Limited quantitative evidence (2 studies); no replication across independent cohorts.	(16, 41)
Coping/hopelessness → HRQOL	1	Serious	Not applicable	Not serious	Serious	Suspected	Very low	Large cohort but single evidence source and construct/outcome overlap.	(57)
Iron deficiency → poorer physical HRQOL	1	Serious	Not applicable	Not serious	Serious	Suspected	Very low	Single biomarker cohort; association was for severe deficiency and physical recovery.	(20)

Ratings apply only to the specified quantitative bodies of evidence. Downgrade reasons are documented in the rationale column and in the factor-specific footnotes below. Participant totals were checked for overlapping cohorts; no overlapping cohorts were identified among the studies contributing to each graded body of evidence. a Risk of bias downgraded due to observational designs and incomplete covariate adjustment. b Inconsistency downgraded where direction or magnitude varied across HRQOL domains or follow-up phases. c Indirectness downgraded where exposure definitions or outcome instruments differed substantially. d Imprecision downgraded when the body of evidence consisted of 2 or fewer studies or wide confidence intervals. e Publication bias suspected for bodies of evidence supported by a single study or limited research groups.

### 3.3 HRQOL among ICU survivors

Across included studies, HRQOL was frequently lower than population norms and, where measured, pre-illness or pre-admission levels. However, baseline HRQOL was not consistently measured, and comparisons with population norms cannot separate pre-existing impairment from changes associated with critical illness. Physical functioning, role functioning, activities of daily living, pain, fatigue, mental health, social functioning, and general health were commonly affected. Early, intermediate, and extended follow-up findings were interpreted separately because recovery is non-linear and associations may vary over time.

From PICS perspective, reduced HRQOL is primarily driven by long-term impairments across physical, psychological, and cognitive domains. Physical sequelae include muscle weakness, ICU-acquired frailty, pain, respiratory dysfunction, fatigue, and reduced activities of daily living. Psychological impairments commonly involve anxiety, depression, post-traumatic stress disorder (PTSD), and sleep disturbances, while cognitive deficits include memory impairment, attention deficits, and reduced executive function. Collectively, these multidimensional impairments significantly compromise functional recovery, social reintegration, and overall quality of life.

### 3.4 Synthesis of factors influencing HRQOL

Based on the Wilson and Cleary model (7), integrated with the PICS (8) and ICF frameworks (14), determinants were synthesized into six core domains. PICS was not treated as an independent determinant of HRQOL; it was used as an overarching clinical framework for organizing new or worsening physical, psychological, and cognitive impairments after critical illness. The individual manifestations of PICS were classified within their corresponding symptom and functional domains.

First, associations between demographic or socioeconomic factors and HRQOL varied across domains and adjustment strategies. Older age and lower educational attainment showed the most frequently reported adverse directions, whereas evidence for sex, income, marital status, and employment was less consistent or potentially overlapped with HRQOL outcomes such as social participation.

Second, pre-existing health status and acute illness characteristics were associated with subsequent HRQOL. Chronic disease burden, frailty, disability, and lower pre-admission HRQOL were among the strongest predictors of poor long-term outcomes in studies that measured them. APACHE II and SOFA scores, organ failure, sepsis, malignancy, trauma, severe COVID-19, and cardiac arrest were also associated with poorer outcomes. Because baseline health influences both ICU exposure and later HRQOL, associations attributed to critical illness or ICU treatment may be partly confounded when pre-admission status is absent or inadequately adjusted.

Third, ICU exposure and hospitalization factors included mechanical ventilation and its duration, tracheostomy, ICU and hospital length of stay, sedation, delirium, vasoactive therapy, blood transfusion, renal replacement therapy, and ECMO. These factors were associated with subsequent HRQOL in some studies, but their independent contribution was not consistent across populations and adjustment strategies. Accordingly, they should be interpreted as prognostic markers or correlates rather than established causes.

Fourth, ICU-acquired weakness, frailty, reduced muscle strength, pain, dyspnea, fatigue, and mobility limitation were among the factors most consistently associated with poorer HRQOL. These findings align with the symptom and functional-status components of the Wilson and Cleary model and with activity limitation in the ICF framework, but overlap between functional exposures and physical HRQOL domains should be considered.

Fifth, anxiety, depression, PTSD symptoms, intrusive memories, cognitive impairment, and sleep disturbance were associated with poorer HRQOL in several studies. These problems may interfere with rehabilitation engagement and social participation, but reciprocal relationships, concurrent measurement, and residual confounding limit conclusions about direction or mediation.

Sixth, family and social support, continuity of care, self-management, rehabilitation participation, and return to work were reported as potentially supportive or protective factors. Quantitative evidence was limited for several of these constructs. Qualitative accounts described informational, psychological, and rehabilitation needs after discharge, but these context-specific findings were not interpreted as universal experiences or as validation of quantitative effects.

### 3.5 Qualitative findings

Two eligible sources contributed survivor-centered qualitative evidence: Scheunemann et al. (15) and the qualitative component of Zhang et al. mixed-methods thesis (16). Oxenbøll Collet et al. (17), is a national survey of nursing staff reporting professional perspectives on patient and family engagement; it was retained as supplementary contextual evidence but was not classified as survivor-centered qualitative data. Participants described disrupted independence and social roles, uncertainty during transitions, and unmet informational or rehabilitation needs. These findings were treated as illustrative and context dependent because the evidence base was small and some themes were supported by only one source.

### 3.6 Pathways for integrating theoretical models

**Figure 2:**
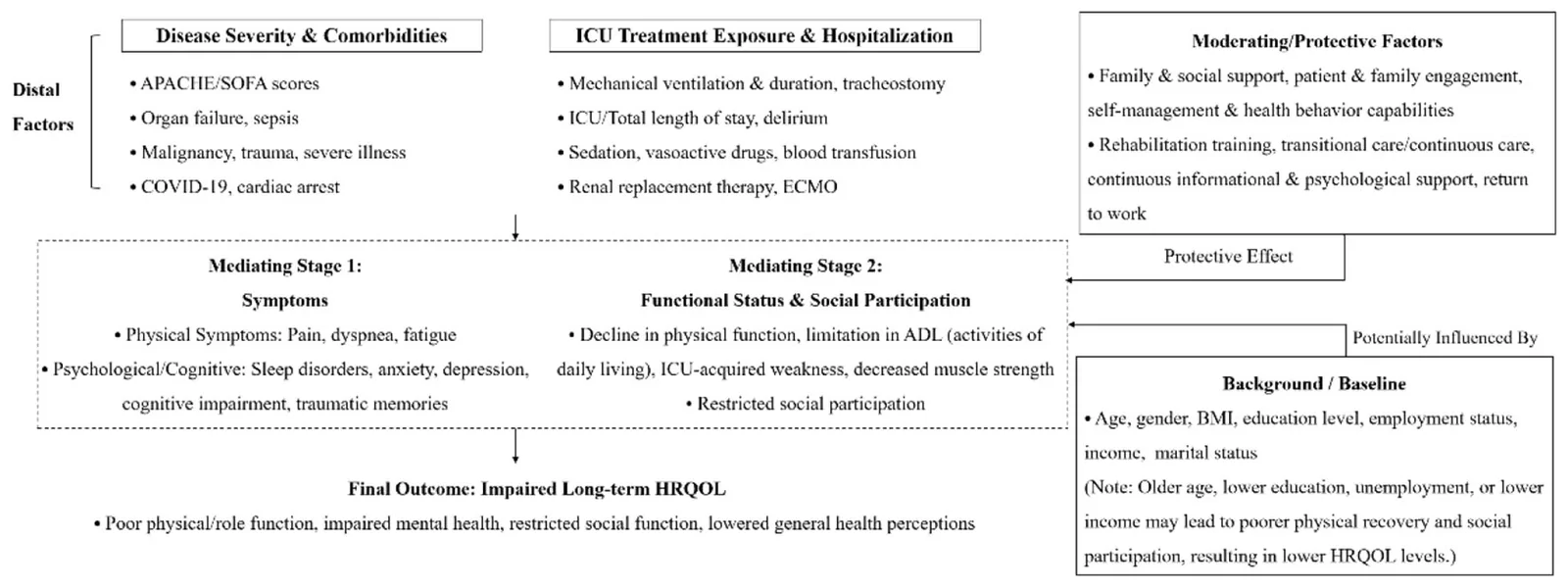
Synthesized conceptual model of determinants of long-term HRQOL after critical illness.

The integrated model places pre-ICU HRQOL, chronic illness, frailty, disability, and socioeconomic context upstream of acute illness. These baseline characteristics may directly influence later HRQOL and may also shape illness severity, treatment exposure, resilience, and access to rehabilitation. Acute illness and ICU exposures were associated with symptom burden, including pain, dyspnea, fatigue, sleep disturbance, anxiety, depression, and cognitive impairment. Functional status and participation may link symptoms with HRQOL, while social support, rehabilitation, and continuity of care may modify recovery. These pathways are conceptual interpretations of predominantly observational evidence and should not be read as proven mediation or causation. The synthesized conceptual model of these determinant pathways is presented in Figure 2.

## 4 Discussion

This mixed-methods systematic review synthesized 53 eligible studies using the Wilson and Cleary model, PICS, and ICF as organizing frameworks. Its contribution is not the rediscovery of established predictors, but a structured reassessment of how confidently reported associations can be interpreted when baseline health, recovery phase, adjustment status, and evidence type are considered together. The review positions pre-existing health upstream, distinguishes statistical associations from experiential evidence, and identifies where findings are consistent, conflicting, complementary, or supported by only one study.

Many studies reported lower HRQOL than population norms, and some longitudinal studies reported improvement between 3 and 12 months. Population-norm comparisons cannot substitute for patient-specific baseline values, and apparent post-ICU deficits may reflect chronic illness, frailty, disability, or socioeconomic disadvantage present before admission. Studies that measured pre-existing disease or baseline function often identified these factors as major predictors of later HRQOL, including Orwelius et al. (18) and Griffith et al. (19). Future cohorts should obtain proxy- or record-based pre-admission HRQOL when prospective measurement is impossible and report both unadjusted change and baseline-adjusted models.

In terms of impairment domains, thematic synthesis revealed a clear three-dimensional pattern of interconnected deficits across physical, psychological, and cognitive domains, consistent with the core clinical phenotype of PICS. At the physical level, reduced muscle strength, ICU-acquired weakness, and severe fatigue are primary proximate determinants of limitations in ADL. At the psychological level, high prevalence of anxiety, depression, sleep disturbances, and PTSD substantially contributes to diminished mental health–related quality of life. At the cognitive level, memory impairment and reduced attention represent a persistent but often underrecognized component of functional decline.

Survivor-centered qualitative evidence from Scheunemann et al. (15). and Zhang et al. (16) provided contextual detail about disrupted priorities, loss of independence or work roles, and unmet informational or rehabilitation needs. Given the small and context-specific evidence base, these themes should be interpreted as illustrative experiences rather than prevalence estimates or universal features.

Within the Wilson and Cleary model (7), biomedical status and treatment characteristics can be treated as distal correlates of HRQOL. Higher illness-severity scores, organ failure, comorbidity burden, malignancy, trauma, and sepsis were associated with poorer outcomes in several cohorts. Yet pre-existing disease and baseline HRQOL may influence both exposure severity and later outcome, creating confounding and regression-to-the-mean concerns. Therefore, the present synthesis cannot determine how much later impairment is attributable specifically to ICU care. Observational associations reported by Lasocki et al. (20) and Puthucheary et al. (21) should be interpreted in light of the covariates included in each model.

Duration of mechanical ventilation, ICU length of stay, and delirium were repeatedly examined as prognostic factors. Several studies reported associations with physical, cognitive, or psychological outcomes, including He et al. (22) and Gamberini et al. (23). However, indication bias and residual confounding are likely because sicker or frailer patients both receive more intensive treatment and have poorer subsequent HRQOL. These exposures should therefore not be described as independent causes unless supported by appropriately adjusted analyses.

Sedation exposure, vasoactive therapy, and delirium were also associated with later psychological or cognitive impairment in some studies (24, 25). Plausible biological mechanisms exist, but the included observational studies do not establish that these exposures caused later deficits. The clinically relevant implication is risk stratification and careful prevention of modifiable complications, not a causal claim from the present synthesis.

The integrated framework proposes, rather than proves, a pathway in which pre-existing health and acute clinical factors are associated with symptoms and functional limitations, which in turn relate to HRQOL. Quantitative studies support the direction and frequency of some associations, whereas qualitative studies describe perceived loss of independence and barriers to participation. Because formal mediation analyses were uncommon, functional status should be considered a hypothesized linking construct rather than a demonstrated mediator. Psychological, cognitive, and sleep problems may also impede rehabilitation, but reciprocal relationships and unmeasured confounding remain possible.

Compared with prior follow-up studies, the present synthesis offers a broader organizing framework rather than a new causal effect estimate. It links frailty, cognition, self-management, rehabilitation, and continuity of care while marking the limits of the evidence. Associations reported by Lin et al. (26), Amacher et al. (27), Li et al. (28), Hao et al. (29), and Yao et al. (30) suggest testable pathways, but mediation and intervention effects require prospective, baseline-adjusted studies.

The convergent synthesis identified complementary evidence from nursing perspectives (Oxenbøll Collet et al. (17) and patient-reported experiences (Zhang) (16). However, Oxenbøll Collet et al. (17) is a quantitative survey of nursing staff rather than a survivor-centered qualitative study; its findings provide professional context but do not constitute survivor perspectives.

Survivors described needs for informational, psychological, and rehabilitation support during transitions. These reports help explain why continuity of care may matter, but the limited qualitative sample does not justify universal language such as a “profound supportive vacuum.” Transferability depends on healthcare system, discharge arrangements, family resources, and access to community rehabilitation.

Continuity of care and timely information may support physical recovery and psychosocial adaptation. However, the available evidence does not establish their effects on self-efficacy, sleep, or return to work. These pathways should be evaluated prospectively using baseline-adjusted designs and clearly defined post-discharge interventions.

## 5 Limitations of the study

Several limitations should be acknowledged. First, populations, case mix, healthcare systems, HRQOL instruments, covariate adjustment, time origins, and follow-up periods were highly heterogeneous. We grouped follow-up into early (< 3 months), intermediate (3–12 months), and extended (>12 months) phases and compared direction and conceptual domains rather than converting scores across instruments; residual incomparability remains. Second, baseline HRQOL, frailty, chronic disease, and disability were inconsistently measured, so ICU-associated estimates may partly represent pre-existing impairment, confounding, or regression to the mean. Third, most evidence was observational; causal and mediation claims are not warranted. Fourth, restriction to Chinese- and English-language reports and exclusion of non-peer-reviewed gray literature (other than indexed theses and dissertations) create risks of publication, language, selective-reporting, and small-study bias. Fifth, survivor-centered qualitative evidence came from only two eligible, context-specific sources; thematic saturation was not claimed. Finally, narrative transformation and framework mapping involve reviewer judgment. Heterogeneity precluded meta-analysis and limited certainty assessment for sparse or non-comparable factors; GRADE judgments were therefore restricted to selected major determinant–HRQOL associations.

## 6 Conclusion

Long-term HRQOL after critical illness is associated with pre-existing health, acute illness, treatment exposure, symptoms, functional status, and environmental support. The most important interpretive point is that chronic disease, frailty, disability, and baseline HRQOL may account for a substantial share of later impairment; post-ICU associations should not automatically be attributed to ICU care. The framework generated here distinguishes statistical associations from lived experience and identifies plausible, testable pathways without asserting causality. Future research should collect pre-admission HRQOL, use standardized recovery windows and core outcome measures, report baseline-adjusted estimates, and test multidisciplinary follow-up strategies in appropriately designed studies.

## Data Availability

The original contributions presented in the study are included in the article/supplementary material, further inquiries can be directed to the corresponding author.
